# High-risk behaviors among adult men and women in Botswana: Implications for HIV/AIDS prevention efforts

**DOI:** 10.1080/17290376.2014.960948

**Published:** 2014-10-08

**Authors:** Mpho Keetile

**Affiliations:** ^a^ PhD Candidate, is affiliated to the Department of Population Studies, University of Botswana, Gaborone, Botswana

**Keywords:** Botswana, implications, high-risk behavior, HIV/AIDS, inconsistent condom use, multiple current partners, Botswana, Conséquences, comportements à risque élevé, Le VIH/sida, l'utilisation du préservatif incompatible, multiples partenaires actuels

## Abstract

The government of Botswana has been spending a lot of money in the prevention, treatment, care and support for HIV/AIDS patient for decades. This paper uses data from the third Botswana AIDS Impact Survey (BAIS III) to explore high-risk behaviors of adults and how they affect government efforts to stop the spread of HIV/AIDS. The objective of this paper is to fill in the gap on the assessment of high-risk behaviors associated with HIV/AIDS and their implications on HIV/AIDS prevention efforts. A nationally representative sample of 10,159 men and women aged 20–64 years who had successfully completed the BAIS III individual questionnaire were used in the study. Both descriptive and binary logistic regression analyses were used for analysis. Crude odds ratios were obtained from gross effects model while adjusted odds ratios (AOR) were obtained from the net effects model. Statistically significant association was observed between multiple current partners and alcohol consumption (AOR = 1.5), drug abuse (AOR = 1.7), transactional sex (AOR = 2.6) and intergenerational sex (AOR = 1.07). Furthermore, statistically significant association was seen for inconsistent condom use and having tested for HIV (AOR = 1.5). These results show a worrying tendency that despite government's efforts to stop the spread of HIV/AIDS, adults in Botswana continue to indulge in high-risk behaviors. Therefore, any programs and policies on HIV/AIDS should first target these high-risk behaviors.

## Résumé

Le gouvernement du Botswana a passé beaucoup d'argent dans la prévention, soins et soutien pour les patients VIH/sida depuis des décennies. Cet article utilise les données de la troisième étude d'Impact de sida Botswana [BAIS III] pour explorer les comportements à risque élevé d'adultes et de leurs effets sur les efforts du gouvernement pour enrayer la propagation du VIH/sida. Cet article vise à combler le vide sur l'évaluation des comportements à risque élevé lié avec le VIH/sida et leurs répercussions sur les efforts de prévention du VIH/sida. Un échantillon national représentatif de 10159 hommes et femmes âgées de 20 à 64 ans, qui avaient terminé avec succès le questionnaire individuel BAIS III ont été utilisés dans l'étude. Analyses descriptive et binaire de régression logistique ont été utilisées pour l'analyse. Brut odds ratio (OR) ont été extraites de modèle à effets brut tout en probabilités ajustées rapports (AOR) ont été obtenus par le modèle des effets nets. Association statistiquement significative a été observée entre les multiples partenaires actuels et la consommation d'alcool (AOR = 1.5), abus de drogues (AOR = 1.7), relations sexuelles transactionnelles (AOR = 2.6) et le sexe entre les générations (AOR = 1.07). En outre, association statistiquement significative a été vu pour l'utilisation du préservatif incompatible et après avoir testé pour le VIH (AOR = 1.5).Ces résultats montrent une tendance inquiétante que, malgré les efforts du gouvernement pour enrayer la propagation du VIH/sida, adultes au Botswana continuent de se livrer à des comportements à risque élevé. Donc des programmes et des politiques sur le VIH/sida doivent tout d'abord cibler ces comportements à haut risque.

## Introduction

Botswana is one of the countries which have the highest HIV prevalence in the world, with an estimated 24% of adults in ages 15–49 infected (UNAIDS 2006). Previous studies in sub-Saharan Africa have shown that alcohol use and drug abuse, transactional and intergenerational sex, inconsistent use of condoms and multiple sexual partnerships are common high-risk behaviors associated with HIV prevalence (See Fritz, Woelk, Bassett, McFarland, Routh, Tobaiwa, *et al.* 2002; Mnyika, Klepp, Kvale & Ole-King'ori 1997; Simbayi, Kalichman, Jooste, Mathiti, Cain, Cherry, *et al.* 2004). Having sexual intercourse under the influence of alcohol or drugs has been associated with increased HIV prevalence (Mnyika, Klepp, Kvale & Ole-King'ori 1996). Alcohol consumption and taking drugs has specifically been associated with a greater likelihood of non-condom use during sexual intercourse (Morrison, Sunkutu, Musaba & Glover 1997; Simbayi *et al.* 2004).

Studies looking at health risk behaviors among adults in sub-Saharan Africa have identified non-use of condoms, multiple sexual partnerships and alcohol/drug abuse as key high-risk behaviors responsible for the spread of HIV/AIDS (Fritz *et al.* 2002; Mnyika *et al.* 1996; Simbayi *et al.* 2004). Multiple sexual relations, which are thought to play an important role in the propagation of HIV in Africa (Gregson, Nyamukapa, Garnett, Mason, Zhuwau, Carael, *et al.* 2002), are often among the key causes of HIV prevalence in Botswana. In Botswana, there are few studies which have examined high-risk behaviors of adults and none has assessed what implications these high-risk behaviors have on government efforts to stop the spread of HIV. Studies in Botswana have often been fragmented, merely looking at the factors influencing the spread of HIV/AIDS. Thus, they have lacked a clear-cut approach to holistically examine high-risk behaviors responsible for the spread of HIV/AIDS but have looked at causal factors in isolation.

Proper characterization of the high-risk behaviors responsible for the spread of HIV/AIDS in Botswana is very critical, especially because HIV prevalence rate had increased by 2.4% in the last AIDS impact survey. This is so because HIV/AIDS burden has heavily impacted on the economic and social lives of Batswana and its causes whether direct or indirect should be averted by all means through relevant and appropriate behavioral change policies and programs. This paper sets out to assess the following; (i) The high-risk behaviors associated with HIV/AIDS prevalence and (ii) the implications of the selected high-risk behaviors on HIV prevention efforts.

## Theoretical orientation

Several theories have been developed to explain health seeking behavior. The most used models for health seeking behavior include among others; Health Belief Model (HBM), the Theory of Reasoned Action (developed later to the Theory of Planned Behavior), the Pathways models, the Health Care Utilization or Socio-Behavioral Model by Andersen (see Andersen & Newman 1973; Meyer-Weitz, Reddy, Van Den Borne, Kok & Pietersen 2000; Munro, Lewin, Swart & Volmink 2007; Noar 2007 for example). Relationships of variables which are considered relevant for explaining or predicting health seeking behaviors are contained in all these models.

This paper is generally guided by notions of individual and social behavioral models that have generally provided direction in the study of health and sexual risk behaviors and associated outcomes. The individual and social behavioral models have often been used to explain why individuals are willing or not willing to undertake a certain action and why they behave the way they do. Individual behavior models focus on the role of individual characteristics in controlling individual behavior, thus they focus on how individuals control their behaviors and make reasoned actions that impact those decisions (Mberu 2010).

Smith (2003) suggests that individual behavior models focus particularly on psychological and cognitive factors believed to influence individual actions and behaviors. The HBM is an example of dominant individual psychological model, which attempts to explain and predict health behaviors and actions by focusing mainly on the attitudes, beliefs and perceptions of individuals (Rosenstock, Strecher & Becker 1994). The HBM has been used over the years to explore various health actions and behaviors, including high-risk sexual behaviors. The basic argument of HBM is the assumption that an individual's characteristics, perceptions, environment and previous experiences are key factors which shape their actions and perceptions of the risks and severity of the outcomes of their behavior, such as indulging in sexual risk behaviors like inconsistent use of condoms during sexual intercourse; having multiple sexual partners; taking alcohol/drugs; and engaging in transactional and intergenerational sex.

One major strength of individual behavior models such as the HBM is that they can be used to predict individual behavior and hence come up with preventive measures to that particular behavior. However, they are limited in that they overgeneralize contextual forces that may influence individual behavior (Mberu 2010). Furthermore, the models lack in the sense that they do not satisfactorily integrate social and cultural norms and peer influences on people's decisions regarding their health-related behaviors. Moreover, the evaluation of interventions based on HBM have shown consistent disappointing effects on high-risky behavior such as inconsistent use of condoms, hence giving impetus to the criticism that an individual is an inadequate unit of analysis (Auerbach, Wypijewska & Brodie 1994).

Smith (2003) observed that cognitive-behavioral, individual models used to study health risk behaviors are limited and have been complemented by social behavioral models that focus on social and community factors operating independently of individual characteristics. The general view of social models is the view that beyond individual personal and psychological characteristics, social relationships and structural factors constrain people's options for behavioral change. Smith (2003) summarized the factors considered by social models to include social pressures, peer influences, cultural expectations, economic factors affecting resource availability, legal and political structures and political and religious ideologies that restrict individual's options and the flow of information.

Although constructs of social and behavioral models discussed above have not been precisely used in the paper, notions of individual and social behavioral models have been used to understand HIV/AIDS risk behaviors among adult men and women in Botswana and the implications of those behaviors on government's efforts to clutch HIV prevalence. It is assumed that if the government of Botswana cannot augment the existing behavioral change programs, then high-risk behaviors such as inconsistent condom use, multiple sexual partnerships, intergenerational and transactional sex are more likely to be persistent hence frustrating government prevention efforts.

## Data and methods

Data derived from the 2008 Botswana AIDS Impact Survey III (BAIS III) was used for this paper. BAIS III is the third of a series of nationally representative demographic surveys aimed at providing up to date information on the Botswana's HIV and AIDS epidemic. The BAIS collects information on HIV prevalence; HIV incidence and socio-economic, demographic and behavioral factors that have a bearing on HIV transmission, care and support. The 2001 Botswana Population Housing Census provided the sampling frame for BAIS III. This frame comprised the list of all Enumeration Areas (EAs) together with number of households. A stratified two-stage probability sample design was used for the selection of the sample. The first stage was the selection of EAs as primary sampling units selected with probability proportional to measures of size (MOS), where MOS were the number of households in the EAs as defined by the 2001 Population and Housing Census (CSO 2008). All 460 EAs were selected with probability proportional to size. At the second stage of sampling, the households were systematically selected from a fresh list of occupied households prepared at the beginning of the surveys' fieldwork. Overall, 8380 households were drawn systematically (CSO 2008). A total of 8380 occupied households were sampled and 7600 were successfully interviewed during BAIS III, yielding a household response rate of 91%. Within the 7600 completed households 16,992 eligible respondents aged 10–64 years were identified out of whom 15,878 were successfully interviewed, yielding an individual response rate of 93% (CSO 2008). This paper is based on a sample of 10,159 men and women aged 20 years and above who had successfully completed the individual questionnaire at the time of the survey.^1^

## Measurement of variables

This section presents a brief description of the measurement of selected key variables used in the paper.

### Dependent variables

The main dependent variable for this paper is sexual and HIV risk behavior which was measured using two separate but related variables. These are (i) multiple concurrent sexual partners – which was derived from a question which sought to find out the number of current sexual partners that a respondent had at the time of the survey and (ii) condom use inconsistency. Condom use inconsistency was measured by responses to questions that sought to find out if the respondents had always used condoms with three different sexual partners. A composite variable for condom use inconsistency with past three sexual partners was then derived from the three questions, which are as follows: Did you always use condoms with most recent partner in past 12 months; Did you always use condoms with next most recent partner in past 12 months and Did you always use condoms with second most recent partner in past 12 months? All the ‘no’ responses were summed up to denote condom use inconsistency.

### Independent variables

BAIS III had questions that are used as proxy for risky behavior related to HIV/AIDS. These HIV/AIDS risk-related behavioral variables are taking drugs or alcohol, ever had sex with someone 10 years older (intergenerational sex) and ever exchanged/receive gift/money for sex (transactional sex). Age, education, marital status, religion, place of residence and HIV testing were included as control variables, because conceptually, and as shown by a number of studies (see Halperin & Epstein 2007; Mah & Halperin 2008; Morris & Kretzschmar 1997), these variables are likely to have an association with the dependent variables, namely men's sexual and HIV risk behaviors. So, in order to hold constant their likely association with the dependent variable, these variables were included in the net effect regression model, so that the relationship between the independent variables becomes isolated and distinct.

### Statistical analysis

The paper applies both bivariate and multivariate analysis to examine covariates of multiple concurrent sexual partnerships and inconsistency in condom use. Binary logistic regression was used to evaluate the effect of a selected group of independent variables on dependent variables. Statistical Package for Social Sciences (SPSS) logistic regression results are presented as adjusted odds ratios (AOR), together with their significance levels. The results of the logistic regression analysis are presented as crude odds ratios (OR) for gross effects model (Model 1) and AOR for the net effects model (Model 2).^2^ The data were analyzed using SPSS version 21 program.

## Results of the study

### Sample description

Table 1 captures the background characteristics of the sample. The sample comprises predominantly females, accounting to more than half (56.2%). Young adults between ages 20 and 29 years account for 38% of the sample, followed by adults aged 40 years and above (32.9%), while individuals between 30 and 39 years account for 28.3%. A significant proportion of respondents had secondary education (42.7%), while 25.4% had primary education, just 17.7% had tertiary education or higher and 14% are those with no education. Over a third (38.9%) of respondents stayed in rural areas, while 32.2% and 28.9% stayed in urban villages and urban areas, respectively. Forty-five percent of respondents in the sample were never married; more than one quarter (25.9% and 29%, respectively) were married and in cohabiting relationships, respectively, while the balance (0.4%) constituted those who were once married (i.e. divorced, widowed or separated from their spouse). Almost 7 out of 10 (69.9%) respondents were Christians; while 5% were of other non-Christian denominations while a quarter (25.2%) of respondents identified themselves as not belonging to any religion.

**Table 1.** Sample background characteristics.

Variable	Number (*N* = 10,159)	Percent
*Sex*		
Male	4446	43.8
Female	5713	56.2
*Age*		
20–29	3934	38.7
30–39	2879	28.3
40+	3346	32.9
*Education*		
No education	1436	14.1
Primary	2579	25.4
Secondary	4341	42.7
Tertiary or higher	1803	17.7
*Residence*		
Urban	2933	28.9
Urban villages	3273	32.2
Rural areas	3953	38.9
*Marital status*		
Never married	4541	44.7
Married	2628	25.9
Living together	2951	29.0
Once married	39	0.4
*Religion*		
Christian	7103	69.9
Other non-christian	492	4.8
No religion	2564	25.2

### Percentage distribution of high-risk behaviors associated with HIV/AIDS

Table 2 shows the percentage of men and women who were involved in high-risk behaviors associated with HIV/AIDS. The results indicate that 1 in every 10 (10.1%) respondents reported that they currently have more than one sexual partner. More than one quarter (28.4%) of respondents indicated that they had used condom inconsistently with three different partners, while more than two-fifths (46.8%) said that they had ever taken alcoholic drink. A small proportion (3.7%) of respondents indicated that they had ever taken drugs, or had received/exchanged gifts/money for sex in the last 12 months prior to the survey (1.8%). Meanwhile, more than one-fifth (23.4%) of respondents said that they had ever had sex with partner 10 years older or younger than them. An encouraging sign though is that, almost three quarters (74.4%) of the respondents had ever been tested for HIV/AIDS.

**Table 2.** Selected key high-risk behavioral variables.

Variable	Number (*N* = 10,159)	Percent
*Currently how many partners do you have?*		
One or none	9125	89.9
Multiple partners	1034	10.1
*Inconsistent condom use index*		
Consistent	7272	71.6
Inconsistent	2887	28.4
*Have you ever taken an alcoholic drink?*		
Yes	4755	46.8
No	5404	53.2
*Have you ever taken drugs?*		
Yes	373	3.7
No	9786	96.3
*Have you ever been tested for HIV?*		
Yes	7559	74.4
No	2600	25.6
*In the last 12 months have you ever received/exchanged gifts/money for sex?*		
Yes	141	1.8
No	10,018	98.2
*Have you ever had sex with a partner 10 years older or younger than you?*		
Yes	2376	23.4
No	7783	76.6

### Inconsistent condom use and background variables

Table 3 shows the percentage of men and women who had not used condoms consistently with three different sexual partners in the past 12 months leading to the survey by selected background characteristics. These results indicate a significant association between inconsistency in condom use with past three partners and background characteristics of respondents. For instance, slightly more females (50.8%) than males (49.4%) reported inconsistent condom use with three different partners, while inconsistency in condom use was also high among respondents aged 40 years and above (40.5%) than among other age groups. The results also indicate that, more than one-third (35.2%) of respondents with secondary education reported inconsistent condom use, while over one quarter (29.8%) and one-fifth (23.4%) with no education and primary education, respectively, reported inconsistent use of condoms. When looking at place of residence inconsistency in condom use was more pronounced in rural areas (46.8%) than in urban areas (22.1%) and rural villages (31%), respectively. Atypically, inconsistent condom use was more pronounced among respondents who are not married (43.3%), than those who are married (28.5%) and living together (27.9%). Furthermore, Christian respondents reported inconsistent condom use more than respondents of other religions and respondents who had no religion.

**Table 3.** Percentage of respondents who reported inconsistent condom use with past three partners and those who had reported multiple sexual partners in the past 12 months leading to the survey by selected background characteristics.

Variable	% Inconsistent condom use	Total	% Multiple sex partners	Total
*Sex*				
Male	49.2*	1421	64.2*	664
Female	50.8	1466	35.8	370
*Age*				
20–29	35.8*	1034	61.8*	639
30–39	23.8	687	26.3	271
+40	40.5	1166	11.9	124
*Education*				
No education	23.4*	677	0.6*	6
Primary	29.8	861	14.8	153
Secondary	35.2	1018	60.5	625
Tertiary or higher	11.3	331	24.2	250
*Residence*				
Urban	22.1*	639	36.3*	376
Urban villages	31.0	896	33.1	342
Rural areas	46.8	1352	30.6	316
*Marital status*				
Never married	43.3*	1251	58.5*	605
Married	28.5	822	11.9	123
Living together	27.9	806	29.6	306
Once married	0.3	8	0	0
*Religion*				
Christian	63.7*	1835	64.5*	667
Other non-christian	6.3	181	6.3	65
No religion	30.0	871	29.2	302
*Overall*	*28.4%*	*2887*	*10.1%*	*1034*

*Statistically significant at *P* = .05.

### Logistic regression results showing the association between selected risk behaviors and inconsistent condom use

Table 4 results show the association between selected risk behaviors and inconsistent condom use. Model 1 presents the gross effects results for each selected risk behavior variable on inconsistent condom use and model 2 introduces the net effects of risk behavior variables on inconsistent condom use and it is at this stage that control variables are introduced.

**Table 4.** Logistic regression results showing the likelihood of association between selected risk behaviors and inconsistent condom use.

	Model 1		Model 2	
Variable	Exp (*B*)	Sig	Exp (*B*)	Sig
*Have you ever taken an alcoholic drink?*				
Yes	1.959	0.000	1.081	0.184
No	1.000		1.000	
*Have you ever taken drugs?*				
Yes	2.143	0.000	0.776	0.084
No	1.000		1.000	
*Have you ever been tested for HIV?*				
Yes	1.802	0.000	1.510	0.000
No	1.000		1.000	
*In the last 12 months have you ever received/exchanged gifts/money for sex?*				
Yes	1.000		1.000	
No	2.302	0.000	1.136	0.557
*Have you ever had sex with a partner 10 years older or younger than you?*				
Yes	1.392	0.000	0.953	0.457
No	1.000		1.000	
*Sex*				
Male			1.788	0.000
Female			1.000	
*Age*				
20–29			1.748	0.000
30–39			1.392	0.000
40+			1.000	
*Education*				
No education			0.532	0.931
Primary			1.349	0.000
Secondary			0.951	0.516
Tertiary or higher			1.000	
*Residence*				
Urban			1.000	
Urban villages			1.105	0.134
Rural areas			0.800	0.001
*Marital status*				
Never married			1.000	
Married			0.313	0.000
Living together			0.598	0.000
Once married			0.531	0.451
*Religion*				
Christian			1.000	
Other non-christian			0.830	0.161
No religion			1.009	0.900

Model 1 results indicate that individuals who had ever taken alcohol were 1.9 times (OR = 1.959) more likely to report inconsistent condom use compared to those who had never taken alcohol. However, when control variables are introduced in the model, the effect of having taken alcohol on inconsistency of condom use diminishes. Similarly, model 1 results indicate that individuals who had ever taken drugs were two times (OR = 2.143) more likely to report inconsistent condom use, compared to those who had never taken drugs, while the effect of having taken drugs on condom use inconsistency diminishes when control variables are included in model 2. Meanwhile, having ever tested for HIV/AIDS was associated with 51% (AOR = 1.510) increase in the odds of having used condoms inconsistently with the past three partners. No significant association was observed between transactional sex, intergenerational sex and inconsistency of condom use.

Moreover, the results indicate that men were 1.8 times (AOR = 1.788) more likely to report inconsistency of condom use with their past three partners when compared to women, while individuals aged 20–29 years (AOR = 1.748) and 30–39 years (AOR = 1.349) were more likely to report inconsistent condom use compared to those aged 40 years and over. Having primary education was associated with 35% increase (AOR = 1.349) in the odds of having not used condoms consistently with past three partners. Married individuals (AOR = 0.313) and those in cohabiting unions (AOR = 0.598) were less likely to report inconsistency in condom use compared to individuals who are never married. No significant association was observed between religion of respondent and inconsistency in condom use.

## Multiple current partners and background variables

Table 3 also shows the percentage of men and women who had multiple current sexual partners by selected background characteristics. The results indicate that almost two-thirds (64.2%) of those who had multiple current partners were men, and individuals aged 20–29 years (61.8%) was the group which reported multiple partners than other age groups. Multiple partnerships were also common among respondents with secondary education (60.5%), those who were never married (58.5%) and professed the Christian religion (64.5%). Meanwhile, when looking at residence, multiple partners were evenly distributed across various residential areas, even though they were slightly higher in urban areas (36.3%), followed by urban villages (33.1%) and rural areas (30.6%).

### Logistic regression results showing the association between selected risk behaviors and multiple current partners

Table 5 results show the association between selected risk behaviors and multiple current partners. Model 1 presents the gross effects results for each selected risk behavior variable on multiple current partners and model 2 introduces the net effects of risk behavior variables on multiple current partners and it is at this stage that control variables are introduced. The results show that respondents who had ever taken alcohol were 1.5 times (AOR = 1.459) more likely to report multiple current partners compared to those who had never taken alcohol. Having ever taken alcohol was significantly associated with 72% (AOR = 1.719) increase in the odds of having multiple current partners. Moreover, respondents who had received/exchanged gifts/money for sex in the past 12 months leading to the survey were 2.6 times (AOR = 2.651) more likely to report multiple sexual partners compared to those who had never received/exchanged gifts/money for sex. Having ever tested for HIV/AIDS was associated with 50% (AOR = 0.503) decline in the odds of having multiple current sexual partners. Meanwhile, no significant association was observed between intergenerational sex and multiple current sexual partners.

**Table 5.** Logistic regression results showing the likelihood of association between selected risk behaviors and having multiple current partners.

	Model 1		Model 2	
Variable	Exp (*B*)	Sig	Exp (*B*)	Sig
*Have you ever taken an alcoholic drink?*				
Yes	2.806	0.000	1.459	0.000
No	1.000		1.000	
*Have you ever taken drugs?*				
Yes	3.653	0.000	1.719	0.000
No	1.000		1.000	
*Have you ever been tested for HIV?*				
Yes	0.887	0.108	0.503	0.000
No	1.000		1.000	
*In the last 12 months have you ever received/exchanged gifts/money for sex?*				
Yes	1.000		2.651	0.000
No	3.657	0.000	1.000	
*Have you ever had sex with a partner 10 years older or younger than you?*				
Yes	0.490	1.052	1.072	0.420
No	1.000		1.000	
*Sex*				
Male			1.431	0.000
Female			1.000	
*Age*				
20–29			0.665	0.000
30–39			0.532	0.000
40+			1.000	
*Education*				
No education			0.431	0.121
Primary			0.361	0.000
Secondary			0.680	0.000
Tertiary or higher			1.000	
*Residence*				
Urban			1.000	
Urban villages			0.627	0.000
Rural areas			0.537	0.000
*Marital status*				
Never married			1.000	
Married			0.173	0.000
Living together			0.533	0.000
Once married			0.531	0.451
*Religion*				
Christian			1.000	
Other non-christian			0.977	0.889
No religion			0.903	0.237

The results also show that men were 1.4 times (AOR = 1.431) more likely to report multiple current partners than women, while individuals aged 20–29 years (AOR = 0.665) and 30–39 years (AOR = 0.532) were less likely to report multiple current partners compared to those aged 40 years and over. Furthermore, individuals who had primary education (AOR 0.361) and secondary education (AOR = 0.680) were less likely to have multiple current sexual partners compared to those with tertiary or higher education. Similarly, individuals who reside in urban villages (AOR = 0.627) and rural areas (AOR = 0.537) were less likely to have multiple current sexual partners compared to those who reside in urban areas. Moreover, married respondents were 83% (AOR = 0.173) and those in cohabiting unions were 47% (AOR = 0.533) less likely to have multiple current sexual partners compared to those who were never married.

## Implications of sexual and HIV risk behaviors on HIV/AIDS prevention efforts

Overall, the results show that risk behaviors leading to HIV/AIDS prevalence are still common in Botswana. The persistence of such behaviors signals the need for target-specific behavioral intervention programs. The implications of high-risk behaviors are not only social but also economic. In response to the enormous challenge of HIV/AIDS, Botswana developed and implemented a series of response plans. A short-term plan was developed soon after the identification of the first case and the focus was on HIV diagnosis and information, education and communication (National AIDS Coordinating Agency 2009). Botswana has over the years mounted a broad multi-sectorial response to HIV/AIDS encompassing both health sector-based and non-health sector-based HIV/AIDS interventions (National AIDS Coordinating Agency 2009). These interventions have been scaled up and very high coverage rates attained. For example, antiretroviral treatment (ART) has been scaled up from an initial 4 sites, and its coverage was estimated to about 82% of all estimated patients in need of ART in 2009 (National AIDS Coordinating Agency 2009).

Routine testing and counseling has been made available and functional in all health facilities with around 150,000 patients tested annually (Ndwapi, Grignon, Buzwani, *et al.* 2012). Prevention of mother-to-child transmission (PMTCT) services have been scaled up to all (634) health facilities with antenatal care and maternity in the country, the uptake of PMTCT is over 85%. Mother-to-child transmission of HIV has been reduced from an estimated 40% without PMTCT to about 4.8% (Ndwapi *et al.* 2012). The National AIDS Council recently recommended safe male circumcision as an additional HIV prevention strategy in the Country. Clearly, these commendable efforts together with other prevention efforts have contributed to the reduction of HIV infection rates in the country. However, the HIV infection rates are still very high. There is perhaps need for the government to make an informed review of current programs and policies on HIV/AIDS to target high-risk behaviors which are responsible for the prevalence of HIV/AIDS in Botswana.

The various HIV/AIDS programs and policies that the government has instituted have had very serious cost implications. For instance, in 2006 it was estimated that average economic growth will be reduced by 1.5–2.0% a year over the period 2001–2021, resulting in the economy being one-third smaller as a result of HIV/AIDS than it would have been otherwise (Econsult Botswana 2006). This negative impact results from reduced labor force growth, a younger labor force, reduced productivity and reduced investment due to HIV/AIDS. HIV/AIDS has had a substantial impact on the government budget, especially under the current scenario of nationwide provision of free ART. The total cost in 2006 was estimated at P1 billion (at 2004/2005 prices), which was equivalent to approximately 6% of government spending. These costs include health-care costs relating to in-patients, ambulatory patients and the ART program, as well as related costs such as home-based care, prevention activities, other HIV/AIDS programs, care of orphans and vulnerable children and additional old aged pensions. The cost of ART drugs is the largest single component of overall costs (World Bank 2012).

The crucial aspect of the fiscal dimension of HIV/AIDS is the persistence of the costs incurred by the impact of and the response to HIV/AIDS. Even taking into account that the HIV/AIDS response in Botswana has been financed partially through external support, and that its fiscal context is relatively kind (though with difficult challenges lying ahead), estimates indicate that the impacts of and the response to HIV/AIDS represent an extraordinary fiscal challenge. The prevalence of high-risk behaviors presents very serious implications on the government's efforts to reduce the prevalence of HIV/AIDS in the country. While the government is trying enthusiastically to halt the prevalence of HIV/AIDS, the continued prevalence of high-risk behaviors renders such efforts regressive. Not only do high- risk behaviors associated with HIV/AIDS render government prevention efforts retrogressive but they also have some cost implications on health expenditure related to HIV/AIDS in the country. Several programs and policies aimed at the reduction of HIV/AIDS prevalence in the country are rendered ineffective by risk behaviors, such as alcohol consumption, drug abuse, intergenerational sex and transactional sex, which ultimately lead to sexual and HIV risk behaviors such as multiple concurrency and inconsistent condom use.

## Discussion and conclusions

While the government of Botswana is fighting hard to combat HIV/AIDS, it is clear that high-risk behaviors are still prevalent among adult men and women in Botswana. The continued occurrence of such behaviors has adverse implications for HIV/AIDS prevention efforts. Overall, the results of this study indicate a positive association between multiple current partnerships and alcohol consumption, drug abuse, transactional sex and HIV testing. Meanwhile, the association between condom use inconsistency and alcohol, consumption, drug abuse, intergeneration sex and transactional sex diminished with the introduction of control variables. However, a positive association was observed between HIV testing and inconsistency in condom use. Thus, individuals who have tested for HIV/AIDS showed high propensity of inconsistent condom use.

These results are in tandem with what previous research in Africa have found, that alcohol use and abuse are associated with risky sexual behaviors, such as multiple concurrent partners (Coleman 1988; Leclerc-Madlala 2008, 2009; Lewis, J. J., Garnett, G. P., Mhlanga, S., Nyamukapa, C. A., Donnelly, C. A., Gregson, S., *et al.* 2005), sexually transmitted disease prevalence (Luke 2005; Mah & Halperin 2008), HIV incidence (Nkosana & Rosenthal 2007) and HIV prevalence (Leclerc-Madlala 2009; Luke 2005). Results further indicate that almost 1 out of 10 Batswana adult men and women reported multiple sexual partners, and multiple concurrent sexual partnerships were more common among men. The prevalence of multiple current partners among men in Botswana has been associated with men's masculinity (see Letamo & Bainame 1997) because men with many women are culturally viewed as ‘man enough’. Multiple sex partnerships among men are an important risk factor for HIV because intergenerational relationships have been noted to confer additional risk for HIV since young sexually active women (Leclerc-Madlala 2008) indulge in sexual relationships with older men. Moreover, women who are themselves monogamous are put at risk through their husbands or male partners who may have multiple partners.

Intergenerational sexual relations and drug and alcohol abuse, which are thought to play an important role in the propagation of HIV in Africa, were associated with having multiple concurrent sexual partnerships. This is a clear indication that elderly men and women in Botswana who are involved in alcohol abuse are more likely to have sexual partners who are 10 years younger or older than them. Since alcohol is the most common form of substance abuse in sub-Saharan Africa (Gillespie & Greener 2006; Glynn, Caraël, Avert, Kahindo, Chege, Musonda, *et al.* 2001; Hallman 2004; Shelton, Cassell & Adejunji 2005) and has been associated with risky sexual behaviors as described above, it may be one of the most common and potentially modifiable HIV risk factors.

Furthermore, the results of this analysis show that men consistently reported multiple concurrent partnerships when compared to women. This is what sexual ethnographic research discovered (Hallman 2004; Leclerc-Madlala 2008, 2009; Luke 2005; Nkosana & Rosenthal 2007) that the significant drivers of the HIV/AIDS epidemic in Eastern and Southern Africa are male attitudes and behaviors, intergenerational sex, gender and sexual violence and multiple concurrent partnerships in which consistent condom use tends to be low. Throughout, sub-Saharan Africa studies have revealed that women's power to negotiate condom use is often compromised by age disparities and economic dependence (Luke 2005; Nkosana & Rosenthal 2007). Women have reported that they often cannot insist on safe sex practices, since doing so would jeopardize their economic goals in the relationship (Glynn *et al.* 2001; Hallman 2004; Luke 2005). As Shelton *et al*. (2005) and Gillespie and Greener (2006) have found, even when African women are relatively well off many still continue to be at risk. This suggests that there should be deliberate efforts to empower women through education and also to enhance their economic dependence to reduce their risk of exposure to unsafe sex practices.

Generally, the results show that high-risk behaviors leading to HIV/AIDS prevalence are still common in Botswana; hence, the need for the government of Botswana to specifically address them, by coming up with target-specific behavioral intervention programs. There is need to review current behavioral programs and develop new strategies which are aimed at adult men and women in Botswana. It is certainly sensible to emphasize the importance of eliminating remaining information gaps between men and women in order to fight the spread of HIV/AIDS. What drives heterogeneities in behavior for men and women is an open question – one that is crucial for policy-makers to learn more about in order to evaluate expenditures on HIV education campaigns and to ascertain what complementary policies are necessary to successfully encourage risk-reducing sexual behaviors. This should be done by taking into cognizance the social differentials within the population, for instance inconsistent condom use was associated with men, low education, young ages and rural areas residence; hence, any program designed to address this sexual and HIV risky behavior should target specific social groups for effective results.

## Limitations of the study

The use of secondary data limits the scope of this paper to variables within the BAIS III data set. The BAIS III did not collect enough variables on high-risk behaviors to allow in-depth analysis of this topic. Like most demographic surveys, the absence of qualitative data denies this analysis in-depth understanding and explanation of patterns observed in the quantitative analysis. However, despite this limitation, these data provide important insights into the potential effects of the high-risk behaviors men and women in Botswana engage in which may ultimately thwart the government's HIV/AIDS prevention efforts.

## Notes

1. The selection criteria of the sample for this paper were such that every male and female respondent aged between 20 and 64 years were included for analysis. This is so because HV/AIDS prevalence levels are higher among this group. The selection was done using SPSS data selection command.

2. Results of the net effects model have been selected and used for interpretation and discussion since they are more apt than results of the gross effects model.
